# Cerebral Small Vessel Disease in Elderly Patients With Menière’s Disease

**DOI:** 10.1097/ONO.0000000000000034

**Published:** 2023-06-15

**Authors:** Fieke K. Oussoren, Roeland B. van Leeuwen, Tjard R. Schermer, Louise N. F. Poulsen, Joost J. Kardux, Tjasse D. Bruintjes

**Affiliations:** 1 Apeldoorn Dizziness Centre, Gelre Hospital, Apeldoorn, The Netherlands; 2 Department of Otorhinolaryngology, Leiden University Medical Center, Leiden, The Netherlands; 3 Department of Primary and Community Care, Radboud Institute for Health Sciences, Radboud University Medical Center, Nijmegen, The Netherlands; 4 Department of Radiology, Gelre Hospital, Apeldoorn, The Netherlands.

**Keywords:** Meniere’s disease, Cerebral small vessel disease, MRI

## Abstract

**Background:**

Menière’s disease (MD) is an inner ear disease characterized by vertigo attacks, progressive hearing loss, tinnitus, and the sensation of aural fullness. Although the exact pathophysiology of MD is unknown, endolymphatic hydrops is considered to be its histopathological hallmark. It has been suggested that endolymphatic hydrops results from lowered perfusion pressure due to cardiovascular comorbidity. Cardiovascular risk factors can cause cerebral small vessel disease (CSVD), visible on MRI. The presence of CSVD in turn raises the risk of developing a stroke.

**Objectives:**

This study aimed to compare the presence of CSVD and cardiovascular risk factors in elderly patients with MD to a control cohort.

**Methods:**

Patients diagnosed with MD, aged 50 years and older, were retrospectively reviewed and compared with a control cohort. The primary outcome was the difference in CSVD on MRI imaging, which was assessed by the number of white matter hyperintensities using the ordinal Fazekas scale. The secondary outcome was the presence of brain infarctions on MRI.

**Results:**

A total of 111 patients with MD were compared with a control cohort of 111 patients. No difference in the degree of white matter hyperintensities (*P* = 0.890) was found between the MD and control cohort. Brain infarctions were seen in 8 of 111 patients with MD and 14 of 111 patients from the control cohort (*P* = 0.261).

**Conclusion:**

CSVD is not more frequently visible on MRI in elderly patients with MD than in controls. This result does not support hypoperfusion-induced ischemia in the pathophysiology of MD.

In 1861, the French physician Prosper Meniere first described the symptoms of a condition that would later be considered Menière’s disease (MD) (1,2). MD is a disease of the inner ear that causes sudden attacks of severe vertigo, progressive hearing loss, tinnitus, and the sensation of aural fullness (3). At present, the pathophysiology of MD is still not unraveled. Endolymphatic hydrops (EH) is the histopathological hallmark of MD, but the exact cause of this EH remains subject to debate.

Foster and Breeze (4) launched the concept that MD results from a combination of hydrops with a variety of risk factors for cerebrovascular disease. They hypothesized that “EH acts as an intermittent Starling resistor to lower perfusion pressure in the inner ear, which is additive with other causes of lowered perfusion such as cerebrovascular disease.” This intermitting hypo- and hyper-perfusion of the brain would lead to transient ischemia and damage to nerve fibers (4).

The findings of Teggi et al. (5) were in agreement with this hypothesis. They studied the disease progression in a population of elderly MD patients and found that the frequency of vertigo episodes and Tumarkin attacks increases with age (5). As age is the greatest contributor to cerebral small vessel disease (CSVD), elderly patients would be especially prone to vascular involvement in the pathophysiology of MD (6).

If cardiovascular risk factors are more frequently present, it is reasonable to suspect that patients with MD exhibit more CSVD on MRI than the general population (7,8). CSVD can be evaluated on MRI by white matter hyperintensities, brain infarctions, microbleeds, lacunes, and dilated Vircho-Robinson spaces (8–10).

In this study, we analyzed whether cerebral MRI imaging in elderly patients with MD exhibits more white matter hyperintensities and brain infarctions compared with a control cohort of patients suspected of trigeminal neuralgia or vestibular paroxysmia.

## MATERIALS AND METHODS

This retrospective case-control study was performed with information extracted from hospital records of patients who visited the Apeldoorn Dizziness Centre (ADC) at Gelre Hospital in the city of Apeldoorn, The Netherlands. The ADC serves as a tertiary referral center that specializes in the diagnostic and therapeutic workup of dizziness. It is a multidisciplinary center involving the otorhinolaryngology, neurology, and clinical neurophysiology departments. This retrospective cohort study was approved by the institutional review board of Gelre Hospital.

### Cohorts

The study cohort was formed with patients diagnosed with MD between January 2010 and March 2021 at the ADC who received an MRI cerebrum to rule out the presence of an acoustic neuroma. According to the criteria developed by the Bárány Society and American Academy of Otolaryngology-Head and Neck Surgery (AAOHNS), patients were divided into a “probable” and a “definite” MD group (3). In all patients with MD, the vestibular function was assessed by either a caloric test, a video head impulse test (video-HIT), or both.

The control cohort was compiled of patients who either visited the outpatient neurological department with facial pain, suspected of trigeminal neuralgia, or patients who visited the ADC with recurrent episodes of spontaneous vertigo lasting several seconds, suggestive of vestibular paroxysmia. These patients all received an MRI cerebrum to rule out the presence of an intracranial neoplasm and/or detect a neurovascular conflict. The control cohort was matched for gender and age with the MD cohort, with a maximum age difference between MD or control patients of 1 year.

Exclusion criteria were age 49 years or younger and a history of a cerebrovascular accident or transient ischemic attack. If during follow-up the type of dizziness changed and did not meet the aforementioned criteria of MD, these patients were excluded.

### MRI Protocol

An MRI was suitable for radiological assessment of white matter hyperintensities and brain infarctions if at least one sequence of the entire brain, either FLAIR or T2, was available. The cerebral sequence was depicted with a slice thickness of 5 mm. The imaging was performed using a 1.5 Tesla MRI scanner. Thirty-five MRI scans were performed in local hospitals and details of the MRI scanners could therefore not be retrieved.

### Outcomes

The presence of the following cardiovascular risk factors was identified from hospital records in all patients; smoking, hypertension, hyperlipidemia, diabetes, a history of myocardial infarction, and atrial fibrillation. The criteria for hypertension were either having a medical history of physician-diagnosed hypertension or using antihypertensive drugs. The criteria for diabetes were having a medical history of physician diagnosed-diabetes or using antidiabetic drugs. In case no complete medical history could be obtained, the variable was defined as missing. Hyperlipidemia was defined as present when having a medical history of physician-diagnosed hyperlipidemia, when using statins, or having an elevated total cholesterol level of >4.9 mmol/L within a month before or after presentation at our dizziness center.

**FIG 1.**
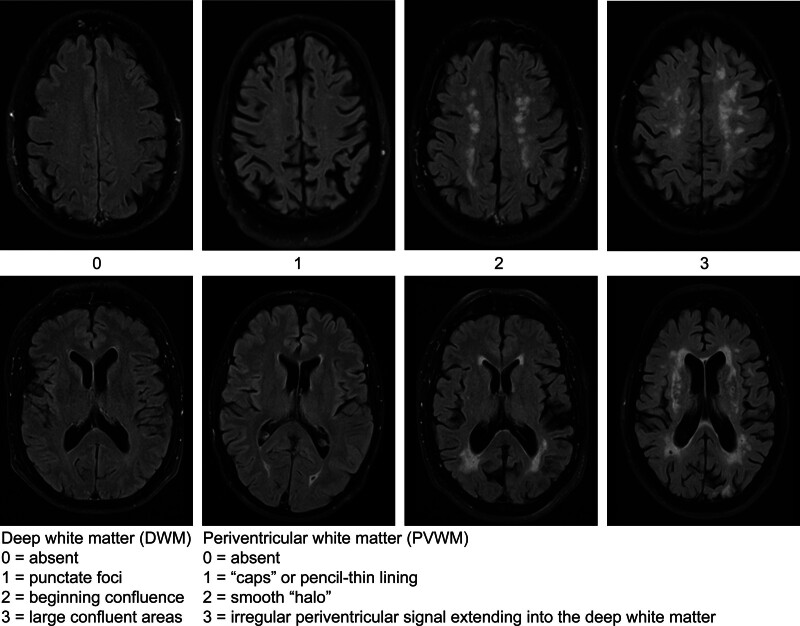
Fazekas scale for MRI imaging. The figure displays hyperintensities in the deep white matter (upper row) and periventricular (lower row) (11).

The primary outcome was the degree of cerebral vascular damage assessed on MRI imaging by measuring the Fazekas score (9). The Fazekas score is a validated diagnostic tool for assessing the severity of white matter hyper-intensities both periventricular and in the deep white matter. The total score is calculated by the sum of the degree of white matter hyperintensities in the periventricular white matter (PVWM) and the deep white matter (DWM). Total scores range from 0 to 6, where 0 means no hyperintensities present, see Figure 1.

The secondary outcome was the presence of brain infarctions on MRI imaging. Brain infarctions were defined by lesions of the brain of at least 3 mm with a cerebrospinal fluid appearance on T2 or FLAIR sequences and differentiable from leukoaraiosis and dilated Virchow-Robinson spaces (12).

### MRI Assessment

MRI imaging was assessed by two radiologists separately, L.P. and J.K. Both radiologists had multiple years of experience in examining MRI imaging of the head and neck. To limit observer bias, the radiologists were blinded to the patient’s study cohort and clinical characteristics. If there was a difference in Fazekas score between the two raters, the following rules were applied. If the inter-rate difference was 1 point, the highest score was applied. If the difference was 2 points or more, the radiologists reviewed the case together until a consensus was reached.

Baseline characteristics, data from diagnostic tests, and MRI ratings were gathered, de-identified, and entered into an electronic database (Castor EDC, Amsterdam, The Netherlands).

### Rater Reliability Testing

Inter- and intra-rater reliability for the two radiologists was assessed for the Fazekas rating scale using 125 MRI imaging of patients who suffered from idiopathic sudden sensorineural hearing loss and 203 controls. All patients were rated by the same raters involved in the present study. Sample size calculation showed that 30 patients had to be rated twice by both raters to evaluate intra-rater reliability. A weighted Cohens’ kappa coefficient was calculated using linear weighting, where the difference between low and high ratings is of equal importance.

### Statistical Analysis

Continuous variables were described using the following summary descriptive statistics: the number of nonmissing values, mean and SD in case of normally distributed data, or median and interquartile range in case of non-normally distributed data. Categorical variables were described using frequencies and percentages.

Statistical testing was performed two-sided at a 0.05 significance level. Differences in the ordinal ranking of the Fazekas scale between the two cohorts were tested using the Mann-Whitney U test for ordinal nonpaired data. The difference in the number of brain infarctions between the cohorts was compared using the chi-square test.

An ordinal logistic regression analysis was performed to compare the primary outcome between both cohorts while adjusting for the potential confounders; gender, age, and the MRI sequence used. To investigate variables within the MD cohort that might influence the Fazekas score, we performed an ordinal regression analysis where we entered age, either a definitive or probable MD diagnosis according to the Bárány criteria, the degree of hearing loss upon diagnosis measured by the high Fletcher index, a vestibular canal paresis defined by an asymmetry in the vestibular function of >20 degrees measured by caloric testing or gain <0.6, measured with video-HIT, and the duration of symptoms before the presentation. Ordinal regression analysis was performed using SPSS version 25. The models used for multivariate analysis were based on forward elimination with a 0.05 significance level.

## RESULTS

### Patient Characteristics

A total of 111 patients with MD were included. The control cohort consisted of 74 patients suspected of trigeminal neuralgia and 37 suspected of vestibular paroxysmia. Patient characteristics are displayed in Table 1. All patients included in the MD cohort had asymmetrical sensorineural hearing loss, but not all patients met the criteria for “definite MD” and were therefore classified as having “probable” MD. One patient had bilateral MD. In the MD cohort, the average duration of symptoms at the time of diagnostic imaging was 5 years (95% CI, 3.4–6.7), with 78 patients having symptoms for over 1 year (70.3%). Sixty-two patients with MD had caloric weakness. In only one patient with MD, the video-HIT was also abnormal. Most patients with MD received a T2 scan of the cerebrum, only 26 of 111 MRIs were assessed using a FLAIR sequence. In the control cohort, 50 of 111 MRIs were assessed using a FLAIR sequence.

**TABLE 1.** Patient characteristics

		MD (n = 111)	Control (n = 111)	Missing	*P* value
Bárány society criteria (%)					
	Definite	84 (75.7)			
	Probable	27 (24.3)			
Age (mean, [SD])		64 (9.4)	64 (9.5)	0	0.423
Gender (%)				0	1.000
	Male	50 (45.0)	50 (45.0)		
	Female	61 (55.0)	61 (55.0)		
Smoking (%)				27	0.027
	Non	74 (77.9)	70 (70.0)		
	Former	3 (3.2)	14 (14.0)		
	Current	18 (18.9)	16 (16.0)		
History of MI (%)		8 (7.2)	5 (4.5)	0	0.569
Hypertension (%)		31 (29.0)	48 (43.6)	5	0.034
Hyperlipidemia (%)		22 (22.2)	30 (27.3)	13	0.427
Diabetes (%)		7 (6.4)	14 (12.6)	1	0.168
Atrial fibrillation (%)		5 (4.5)	2 (1.8)	1	0.280
Anticoagulant use (%)		20 (18.0)	11 (9.9)	0	0.120

Patient characteristics of 111 patients diagnosed with MD and the control cohort of 111 patients, displayed in numbers and percentages. For age, the mean and SD are displayed. The number of missing cases is the sum of missing cases in both cohorts.

MD, indicates Meniere’s disease; MI, myocardial infarction; n: number.

The prevalence of smoking was only slightly higher in patients with MD, whereas significantly more patients in the control cohort were former smokers (*P* = 0.027). Hypertension was more frequently present in the control cohort.

### Rater Reliability Testing (Results Based on a Previously Investigated Cohort)

A total of 328 MRI scans were reviewed by both raters. In 201 cases both radiologists gave the same Fazekas score, the score differed by one point in 103 cases and by 2 or more points in 24 cases. This resulted in a kappa-coefficient of 0.74, suggesting substantial inter-rater reliability.

Thirty subjects were rated twice by each rater, which resulted in a weighted kappa-coefficient of 0.80 and 0.82 for rater 1 and 2, respectively, suggesting an excellent intra-rater agreement for both raters.

### Fazekas Score

**FIG 2.**
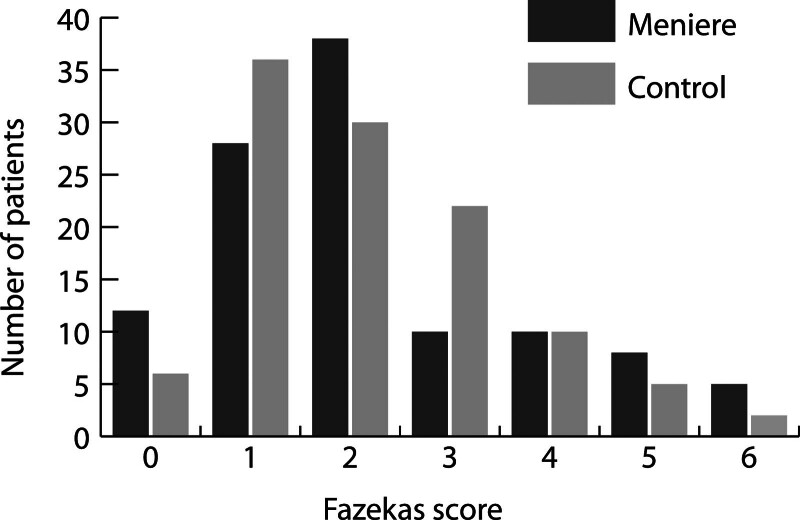
The distribution of Fazekas score among patients with MD and controls. A Mann-Whitney U test was performed to compare the differences in the ordinal variable Fazekas score between both cohorts which demonstrated a nonsignificant result (*P* = 0.968). MD, Meniere’s disease.

Most patients with MD received a Fazekas score of 2 (34.2%), while Fazekas 1 was most frequently present in the control cohort (32.4%). The differences in the distribution of the Fazekas scores in the two cohorts, taking an ordinal ranking into account, were not statistically different (*P* = 0.968); see Figure 2. When we analyzed the Fazekas scores for PVWM and DWM separately, the distribution between both cohorts was not statistically different either, *P* = 0.972 and *P* = 0.958 for PVWM and DWM, respectively.

The univariate ordinal logistic regression model demonstrates that MD did not influence the Fazekas score (ie, odds ratio [OR] = 0.967 [95% CI, 0.605–1.546] for MD patients relative to control patients). In the multivariate analysis, both age and the MRI sequence used for assessment were associated with the Fazekas score, but the MD was not. Within the cohort of MD patients, age and a disease duration of over 1 year at the time of imaging were associated with a higher Fazekas score in the multivariate analysis; see Table 2.

**TABLE 2.** Results of ordinal logistic regression analysis

	Variables	Univariate			Multivariate		
		Odds ratio	*P* value	95% CI	Odds ratio	*P* value	95% CI
A	Meniere cohort	0.967	0.889	0.605–1.546	1.137	0.607	0.697–1.853
	Age	1.109	<0.001	1.078–1.140	1.108	<0.001	1.077–1.140
	Gender (female)	1.376	0.196	0.851–2.195			
	FLAIR sequence	2.128	0.003	1.288–3.518	2.163	0.004	1.285–3.641
B	Age	1.058	<0.001	1.044–1.127	1.092	<0.001	1.050–1.136
	Definite MD	1.355	0.443	0.624–2.941			
	Abnormal caloric testing	0.696	0.166	0.417–1.162			
	Fletcher index	1.013	0.099	0.998–1.028			
	Duration of symptoms^a^	2.384	0.023	1.130–5.029	2.879	0.006	1.345–6.162

a. Outcomes of the univariate (left) and multivariate (right) ordinal regression analysis including all 222 patients. Estimated odds ratio, its significance and 95% CI, of receiving a higher Fazekas score when having female gender, MRI sequence used and the study arm. The reference of interpretation of the odds for the Meniere cohort was the control cohort.

b. Outcomes of a second univariate ordinal regression analysis including only MD patients to evaluate the influence of abnormal caloric testing, the Fletcher index, probable vs definite MD disease and the duration of symptoms on the Fazekas score within MD patients. The reference for interpretation of the odds for the Fletcher index is a 1-point higher Fletcher index.

^a^Duration of symptoms is a binary variable with symptoms existing either shorter or longer than one year, the reference in the regression analysis is symptoms shorter than 1 year.

CI indicates confidence interval, FLAIR, Fluid-attenuated inversion recovery, Sig, significance, MD, Meniere’s disease.

### Brain Infarctions

Brain infarctions were present in 8 patients (7.2%) in the MD cohort and 14 (12.6%) in the control cohort. This difference was not statistically significant (*P* = 0.261).

## DISCUSSION

To date, the exact pathophysiology of MD is not clarified. As a consequence, treatment options are limited and often nonsuperior to the natural disease progression (4,13). Although a vascular cause has been suspected in certain cases of MD, neither the degree of white matter hyperintensities nor the presence of brain infarctions differed between elderly patients with MD and controls in this study.

In 2013, Foster and Breeze (4) presented a hypothesis of hypoperfusion-induced ischemia as the pathophysiological mechanism responsible for MD. They proposed that a Meniere’s attack is caused by three interacting factors. First, preexisting hydrops is necessary, but not sufficient by itself, to cause an attack (4). This is emphasized by the fact that EH is present in all patients with MD, but can also be radiologically diagnosed in up to 31% of the healthy population (14).

Second, inner ear pressure fluctuates heavier in patients with MD than in the healthy population in response to changes in atmospheric pressure, head position, etc. Since cerebral perfusion pressure is determined by arterial pressure, intracerebral fluid pressure, and venous outflow resistance, disturbances in one of these factors can cause hydrops. This hydrops in turn causes intermittent hypo- and hyperperfusion of the vasculature of the ear when the volume capacity is exceeded (4).

Third, the arterial blood supply to the labyrinth is provided by the arteria labyrinthi, while the endolymphatic sac is believed to be supplied by a branch of the external carotid artery (15). There is little collateral circulation, hence the cochlea and vestibule are vulnerable to ischemia by hypoperfusion.

Foster further hypothesizes that “in young individuals, with normal vasculature and normal oxygen levels, the highest pressure reached in the hydropic ear does not exceed the critical perfusion pressure for the tissues and is therefore not sufficient to cause ischaemia” (4). The cardiovascular comorbidity in the elderly makes them more prone to hypoperfusion-induced ischemia in comparison with young individuals, due to their reduced arterial pressure (4). Foster (16) advocated screening all patients with MD for cardiovascular risk factors and modifying their treatments accordingly. So far, the hypothesis proposed by Foster and Breeze (4) is not supported by clinical research.

In recent years, hypoperfusion and ischemia have also been suspected to cause sudden deafness. A recent meta-analysis demonstrated a higher risk of stroke in patients who had experienced sudden deafness compared to healthy controls (17). Also, cardiovascular risk factors were more common in patients with sudden deafness than in the general population (18,19). The recurring nature of vertigo spells in MD differs from the hearing loss in sudden deafness, which has a very low recurrence rate (20). One would expect that, if ischemia in excitatory fibers within the labyrinth causes EH, this would be irreversible and result in persistent vestibular loss of function. Persistent vestibular loss, however, does not occur in all patients and the course of the vestibular function does not correlate with the subjective severity of vertigo attacks (21).

To investigate the plausibility of ischemia in the pathophysiology of MD, one can analyze the correlation between cardiovascular disease and MD. Fazekas et al. and Wardlaw et al. have demonstrated that the cardiovascular risk factors hypertension, hyperlipidemia, age, and cardiovascular comorbidity like myocardial infarction, raise the risk of developing stroke (8–10,22,23). These cardiovascular risk factors are responsible for cerebral small vessel disease, visible on MRI as white matter hyperintensities, microbleeds, silent brain infarctions, and lacunes (23). Our study is the first to evaluate the presence of white matter hyperintensities in an MD population. We did not find a difference in the degree of CSVD between patients with MD and controls. Therefore, our results do not support the hypothesis of Foster and Breeze (4). According to this hypothesis, one would suspect Meniere attacks to be caused by hypoperfusion-induced ischemia in elderly patients with cardiovascular comorbidity. It is likely that this ischemia would not be limited to the vestibule and cochlea, but would also affect other vulnerable areas of the brain with little collateral vascular supply, which should be visible on MRI by the previously mentioned characteristics.

In the present study, none of the cardiovascular risk factors were more frequently present in the MD cohort. However, due to the retrospective design of this study, the prevalence rates of the cardiovascular risk factors are not entirely reliable. So far, Rego et al. (24) were the only ones investigating cardiovascular comorbidity in patients with MD and its association with the course of the disease. They found a statistically significant association between the occurrence of Meniere’s disease attacks and cardiovascular risk factors, with 74% of the population having at least one risk factor. This result, however, was based on a small study population consisting of only 31 patients, without comparison with a control cohort (24).

In agreement with existing literature, Kim et al. (25) recently demonstrated an increased risk of migraine in patients with MD and vice versa. Kim et al. (25) also reviewed the incidence of several cardiovascular risk factors in patients with MD, migraine, and controls. Overall, controls had a higher systolic and diastolic blood pressure, higher blood glucose levels and were more frequently smokers than patients with MD or migraine (25). This nonelevated cardiovascular risk does not correspond with the theory suggested by Forster and Breeze.

Nonetheless, our results do not rule out the possibility of vascular involvement in the pathophysiology of MD through another mechanism. Sarna et al. (26) suggested that, in addition to their shared epidemiological association, migraine and MD may have a similar pathophysiological mechanism. Spreading cortical depression is believed to result in a release of vasoactive neurotransmitters such as calcitonin gene-related peptide and substance P from the trigeminal nerve ganglion, causing vasodilatation, increased vascular permeability and extravasation of plasma (26). This process is believed to be responsible for migraine headaches. Likewise, innervation of the cochlear vasculature by trigeminal nerve stimulation has been described to cause extravasation of fluid in the cochlea (27). This mechanism could theoretically cause EH. The effect of agents blocking the receptors of these vasoactive neurotransmitters and their effect on migraine headaches and generalized cardiovascular disease is currently being investigated (28).

Our study has several limitations due to its retrospective design. First and most importantly, the control cohort was compiled of patients who had an indication for an MRI of the brain, which was to detect a neurovascular conflict of the fifth or eighth cranial nerve. These patients, therefore, cannot entirely be considered healthy controls and might have more cardiovascular comorbidity than healthy subjects. It is unlikely, however, that healthy subjects would have a lower Fazekas score than our control cohort since a mean Fazekas score of 1 has been previously documented in healthy elderly in a similar age category (29,30).

Second, several assumptions were made in the recording of cardiovascular risk factors as described in the methods section and the actual presence of these risk factors in the MD patients and controls is, therefore, not entirely reliable. Also, the duration of disease in patients with MD had a significant range. However, we did not find a correlation between the duration of the disease and Fazekas score.

Third, the MRI sequence that was used for assessment was non-uniform. Usually, an MRI cerebrum is performed in patients suspected of trigeminal neuralgia, while in the case of MD, an MRI of the cerebellopontine angle is performed with only a single sequence of the entire brain, either T2 or FLAIR. In the regression analysis, we did see that the FLAIR sequence correlated significantly to a higher Fazekas score. This might have biased our results since the MRI scans of patients with MD were less frequently assessed using a FLAIR sequence compared with controls. Nevertheless, when we entered the cohort in the multivariate analysis the odds did not alter.

In conclusion, cerebral small vessel disease and cardiovascular risk factors were not observed more frequently in patients with MD than in matched controls. This result does not support the hypothesis of hypoperfusion-induced ischemia in the pathophysiology of MD in elderly patients.

## FUNDING SOURCES

None declared.

## CONFLICT OF INTEREST

None declared.

## DATA AVAILABILITY

The datasets generated during and/or analyzed during the current study are not publicly available but are available from the corresponding author on reasonable request.
